# The Role of Pulmonary Veins in Cancer Progression from a Computed Tomography Viewpoint

**DOI:** 10.1155/2016/1872627

**Published:** 2016-09-22

**Authors:** Chuang-Chi Liaw, Hung Chang, Tzu-Yao Liao, Ming-Sheng Wen, Chih-Teng Yu, Yu-Hsiang Juan

**Affiliations:** ^1^Division of Hemato-Oncology, Department of Internal Medicine, Chang-Gung Memorial Hospital, Chang-Gung University College of Medicine, Taoyuan, Taiwan; ^2^Division of Cardiology, Department of Internal Medicine, Chang-Gung Memorial Hospital, Chang-Gung University College of Medicine, Taoyuan, Taiwan; ^3^Division of Chest, Department of Internal Medicine, Chang-Gung Memorial Hospital, Chang-Gung University College of Medicine, Taoyuan, Taiwan; ^4^Department of Medical Imaging and Intervention and Department of Internal Medicine, Chang-Gung Memorial Hospital, Chang-Gung University College of Medicine, Taoyuan, Taiwan

## Abstract

*Background*. We studied the role of pulmonary veins in cancer progression using computed tomography (CT) scans.* Methods*. We obtained data from 260 patients with pulmonary vein obstruction syndrome (PVOS). We used CT scans to investigate pulmonary lesions in relation to pulmonary veins. We divided the lesions into central and peripheral lesions by their anatomical location: in the lung parenchymal tissue or pulmonary vein; in the superior or inferior pulmonary vein; and by unilateral or bilateral presence in the lungs.* Results*. Of the 260 PVOS patients, 226 (87%) had central lesions, 231 (89%) had peripheral lesions, and 190 (75%) had mixed central and peripheral lesions. Among the 226 central lesions, 93% had lesions within the superior pulmonary vein, either bilaterally or unilaterally. Among the 231 peripheral lesions, 65% involved bilateral lungs, 70% involved lesions within the inferior pulmonary veins, and 23% had obvious metastatic extensions into the left atrium. All patients exhibited nodules within their pulmonary veins. The predeath status included respiratory failure (40%) and loss of consciousness (60%).* Conclusion*. CT scans play an important role in following tumor progression within pulmonary veins. Besides respiratory distress, PVOS cancer cells entering centrally can result in cardiac and cerebral events and loss of consciousness or can metastasize peripherally from the pulmonary veins to the lungs.

## 1. Introduction

Pulmonary veins carry oxygenated blood from the lungs to the left atrium of the heart. There are typically four pulmonary veins: the right superior, right inferior, left superior, and left inferior [1]. Tumor thrombosis from pulmonary veins can enter centrally into the left atrium [2–4] or extend peripherally from the pulmonary vein [4].

Cytokines can be produced by cancer and are involved in the host response to tumor growth, cancer invasion, and metastasis [5]. Cancer cells can pass into the pulmonary vein through lung capillaries and through direct extension [6]. Sienel et al. [7] reported that patients with operable non-small-cell lung cancer had a poor clinical outcome when disseminated cancer cells were found in their pulmonary venous blood. A study conducted by Phillips et al. [8] reported that circulating tumor cells appeared at a significantly higher rate in pulmonary venous blood during primary lung cancer surgery. Hashimoto et al. [9] found that circulating tumor microemboli have thrombotic potential. Our previous article [4] reported that tumor thrombus, coagulation, and cytokines contribute to pulmonary venous obstruction syndrome (PVOS) in cancer patients. We found that cytokines were the main culprit for causing this syndrome. Several inflammatory cytokines have been shown to participate in the progression of the epithelial-to-mesenchymal (EMT) transition, which is associated with cancer metastasis and therapy resistance [10–15].

In the present case series study, we investigate the role of pulmonary veins in cancer progression using computed tomography (CT) scans. The data were obtained from 260 cancer patients with PVOS.

## 2. Methods

### 2.1. Patients

Between January 2005 and December 2015, we conducted a case series study. Data were collected from 1256 patients hospitalized in oncology wards of the Chang-Gung Memorial Hospital. Our data was provided mainly from a single physician. Our focus was on urological cancer: most of the patients had urothelial carcinomas. The data for the 260 cancer patients with PVOS was obtained from the original 1256 patients.

### 2.2. Diagnostic Criteria of PVOS

The criteria for a diagnosis of PVOS included dyspnea symptoms (1), chest X-ray findings (2), and CT findings (3). All three of these criteria points were required to be present in the patient for use in this study [6]. To elaborate, these criteria involve (1) episodes of shortness of breath; (2) chest X-rays that show unilateral or bilateral abnormal pulmonary hilum shadows, with or without the presence of pulmonary edema and/or pleural effusion; and (3) CT scans that demonstrate pulmonary vein thrombosis/tumors, sticking to the outer vein surface with or without lesions. When dyspnea occurred and chest X-rays showed an abnormal hilum shadow, CT scans were used to detect pulmonary vein thrombosis/tumors.

### 2.3. Clinical Investigation

The patients' characteristics usually combined with other thromboembolic complications and paraneoplastic syndromes. This may result from the aggravation of acute respiratory distress by chemotherapy and by other medical or surgical procedures. Acute respiratory distress is also subject to diurnal fluctuations in intensity. Common thromboembolism-associated complications included loss of consciousness and mental change [16, 17], paraneoplastic pain, and iliofemoral venous obstruction or thrombosis. Paraneoplastic pain is defined as a breakthrough pain that occurs in the absence of an identifiable precipitating cause [18]. Cerebral thromboembolic complications and paraneoplastic pain in most patients could only be clinically suspected, due to the difficulty in acquiring a definite diagnosis. Paraneoplastic syndromes included neoplastic fever (tumor-related fever that responds well to the naproxen test) [19] and cachexia syndrome (a weight loss of > 5% within six months, with reduced food intake and muscle wasting) [20].

### 2.4. Laboratory Study

A D-dimer test using enzyme-linked immunosorbent assay (ELISA) was conducted on all patients, and C-reactive protein levels were checked in selected patients. The cutoff D-dimer value was 500 ng/mL. Paraneoplastic syndromes included hypercalcemia (serum calcium levels that exceeded 11 mg/dL) and a leukemoid reaction (peripheral count that exceeded 20,000/*μ*L without evidence of infection or leukemia).

### 2.5. Image Study

We divided the pulmonary lesions into central and peripheral lesions depending on their anatomical position: in lung parenchymal tissue or within pulmonary veins; in the superior or inferior pulmonary veins; and by unilateral or bilateral presence in the lungs. Pulmonary lesions from the CT scans were divided into four subgroups: central parenchymal lung lesions, central thrombotic lesions, peripheral parenchymal lung lesions, and peripheral thrombotic lesions. A central parenchymal lung lesion was defined as a lung parenchymal lesion that did not adhere to the proximal pulmonary vein. A central thrombotic lesion was defined as a thrombus or tumor that was located within the proximal pulmonary vein, near to or extending into the left atrium. A peripheral parenchymal lung lesion was defined as a lesion with a distal-peripheral location to the pulmonary vein. A peripheral thrombotic lesion was defined as a thrombus/tumor lesion located within or along the pulmonary vein. Mixed types were defined as a mixed central (either parenchymal or thrombotic) lesion with a peripheral (either parenchymal or thrombotic) lesion subtype. Obvious cardiac extension was defined as lesions extending into the left atrium, as shown by CT scans. Finally, we defined the presence of nodules to be when nodules were noticed within pulmonary veins, as shown by CT scans or from the lung window.

### 2.6. Therapy

Therapeutic treatments included the subcutaneous injection of low molecular weight heparin (LMWH), either Fraxiparin (GlaxoSmithKline; 3800 IU or 5700 IU daily) or enoxaparin (Sanofi-Aventis; 6000 IU daily), intravenous dexamethasone, intravenous fluids, or furosemide when PVOS occurred along with acute respiratory distress. Further use of chemotherapy, targeted therapy, or hormone therapy depended on the patient's condition. CT scans were obtained from the hospital picture archiving and communication system (PACS).

### 2.7. Statistical Methods

Continuous data (presented as mean ± standard deviation) were used for the D-dimer and C-reactive protein level tests. Survival was calculated as the time from the diagnosis of PVOS to the patient's death. Survival curves were determined using Kaplan-Meier methods.

## 3. Results

### 3.1. Patient Characteristics

Of the original 1256 patients, 260 patients (20%) were determined to have PVOS. PVOS was documented from clinical symptom, chest X-ray, and CT scan findings. The data for these 260 cancer patients (164 men and 96 women; 27–93 years old; median age, 71) was collected for the evaluation of their pulmonary veins.  Table 1 shows the important findings from the PVOS patients. PVOS occurred in patients with various metastatic tumors. 185 patients (71%) had an Eastern Cooperative Oncology Group (ECOG) performance status of 2 or greater. A common association with thromboembolic complications occurred in 171 patients (66%): consciousness disturbance in 124, paraneoplastic pain in 55, and iliofemoral venous obstruction or thrombosis symptoms in 21. Of them, 66 patients had multiple thromboembolic presentations. Of the 124 patients with consciousness disturbance, 18 had angiographic, CT scan or magnetic resonance imaging (MRI) evidence of cerebral infarction. Common association with paraneoplastic syndromes occurred in 131 patients (50%): cachexia syndrome in 105, neoplastic fever in 29, leukemic-like reactions in 19, hypercalcemia in 6, and lactic acidosis in 3. Of these 131 patients, 19 had multiple paraneoplastic syndromes.

### 3.2. Clinical Outcomes

Shortness of breath was the main symptom. Acute respiratory distress (*n* = 260) was aggravated by chemotherapy in 28 patients (11%), or by medical or surgical procedures in 23 patients (9%), and showed diurnal fluctuations in intensity in 35 patients (13%). D-dimer and C-reactive protein levels were checked in all patients. The mean D-dimer value was 3214 ± 2162 ng/mL (ranging from 265 to greater than 10,000 ng/mL). D-dimer values were less than 1000 ng/mL in 32 patients (12%), 1001–3000 ng/mL in 96 patients (36%), 3001–5000 ng/mL in 56 patients (22%), and greater than 5000 ng/mL in 79 patients (30%). The mean C-reactive protein value was 112 ± 94 g/d (it ranged from 0.7 to 384 g/d). 121 patients (93%) had elevated C-reactive protein values.

**Figure 1:**
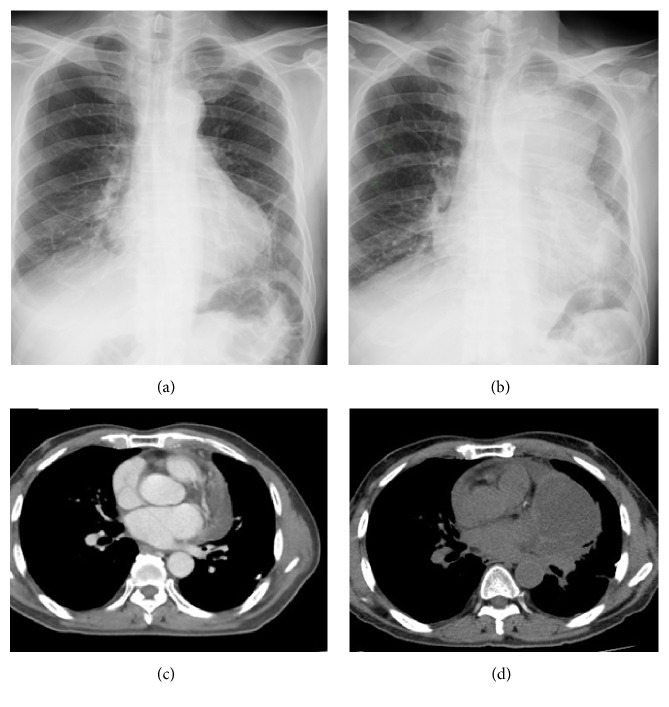
Central parenchymal pulmonary lesions. The patient is a 63-year-old man with renal cell carcinoma who underwent targeted therapy. He felt dyspnea. Chest X-rays were taken at time (a) and 1 month later (b), which shows the rapid progression of the left upper lung mass. A CT scan (c) shows the bulging left superior vein and 1 month later (d) reveals the lung parenchymal mass located outside the left superior pulmonary veins.

**Figure 2:**
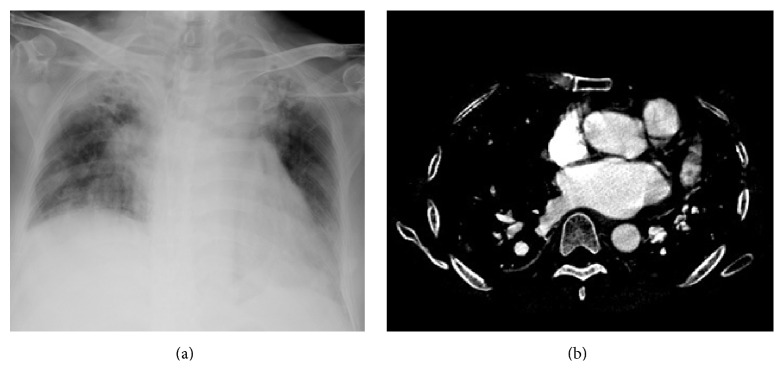
Central thrombus lesions. The patient is a 71-year-old woman with renal pelvis urothelial carcinoma who underwent chemotherapy. She felt dyspnea. A chest X-ray (a) shows bilateral lung hilar lesions. A CT scan (b) reveals bilateral thrombus/tumor located bilaterally within the superior pulmonary veins.

**Figure 3:**
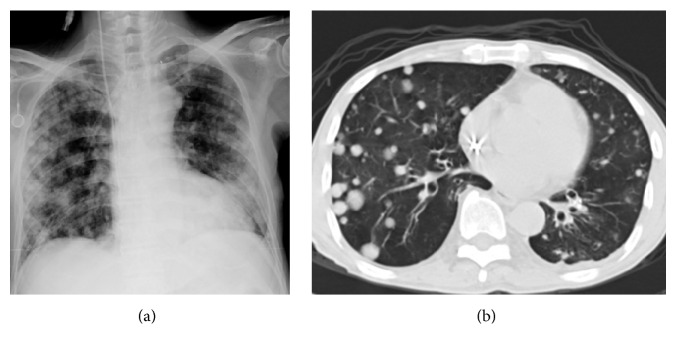
Peripheral parenchyma pulmonary lesions. The patient is a 58-year-old man with bladder urothelial carcinoma who underwent chemotherapy. He felt dyspnea. A chest X-ray (a) shows multiple lung metastases. A CT scan with a lung window (b) shows several nodules located in the inferior pulmonary vein with multiple lung parenchymal metastases.

**Figure 4:**
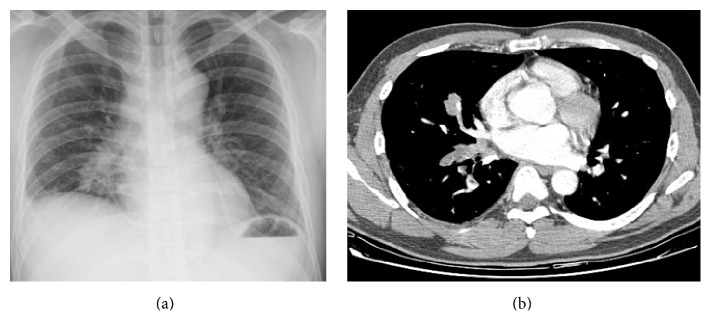
Peripheral thrombotic lesions. The patient is a 41-year-old man with lung cancer. He felt dyspnea. A chest X-ray (a) shows a low right lung lesion. A CT scan (b) shows abnormal tumor/thrombotic lesions within the right superior and right inferior pulmonary veins, with extension into the right lung parenchymal tissue.

**Figure 5:**
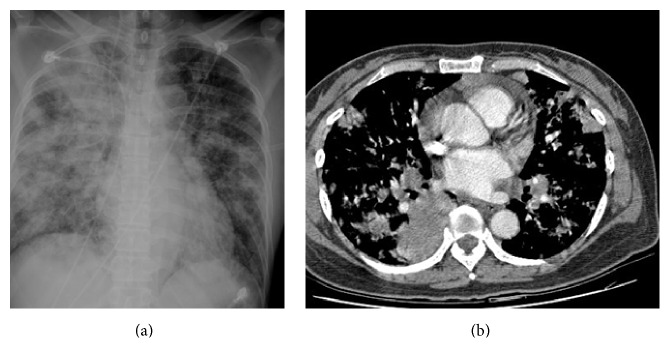
Mixed central and peripheral thrombotic and parenchymal pulmonary lesions. The patient is a 45-year-old woman with colon cancer. She felt dyspnea. A chest X-ray (a) shows a right upper lung mass with multiple lung metastases. A CT scan (b) shows a right upper lung mass next to the right superior pulmonary vein with multiple peripheral lung metastases.

**Figure 6:**
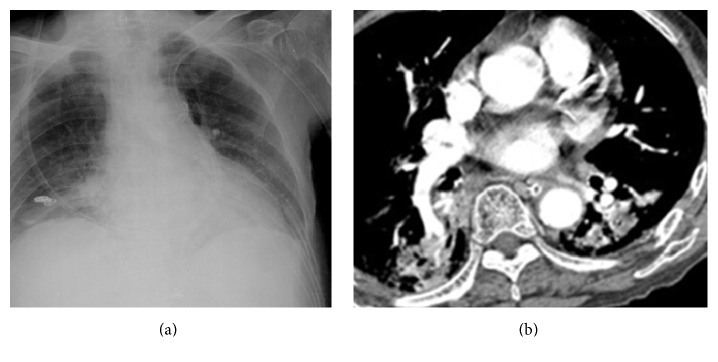
Mixed central and peripheral thrombotic and parenchymal pulmonary lesions. The patient is an 87-year-old women with hepatocellular carcinoma. She felt dyspnea. A chest X-ray (a) shows a low right lung lesion. A CT scan (b) reveals abnormal tumor/thrombotic lesions within the right superior pulmonary vein with extension into the left atrium and into the peripheral lung parenchymal tissue.

**Figure 7:**
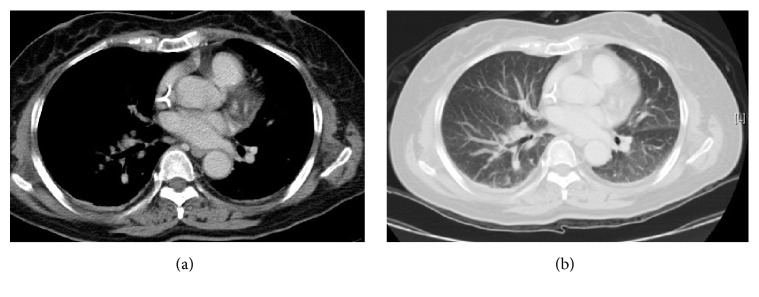
Nodules within pulmonary vein. The patient is a 62-year-old women with lung squamous cell carcinoma who underwent chemotherapy. She felt dyspnea. A chest CT scan (a) and the lung window (b) showed several nodules within her right inferior pulmonary vein.

**Figure 8:**
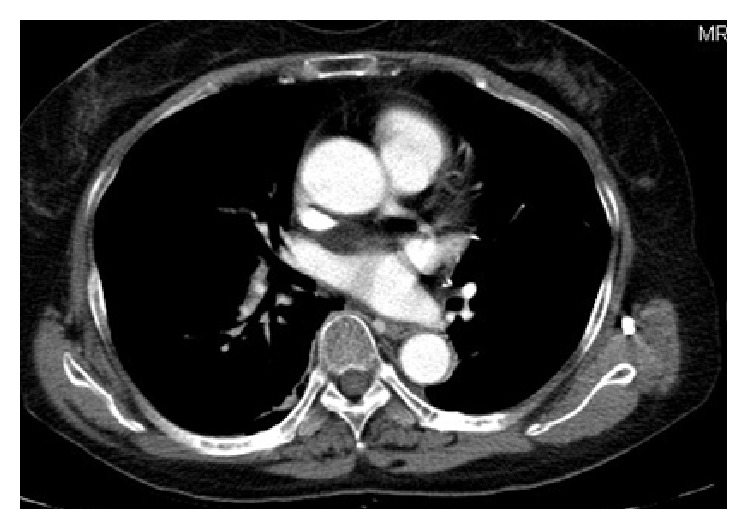
Nodules within pulmonary vein. The patient is a 63-year-old women with adrenocortical carcinoma and with asymptomatic lung metastasis. A chest CT scan shows abnormal nodules and lesions within the right superior pulmonary vein and a tumor/thrombotic lesion in the left atrium and the left superior pulmonary vein.

Table 2 showed chest CT scan findings relating to the pulmonary veins from 260 cancer patients with PVOS. They were categorized by lesion location into the following groups: central location (Figures 1 and 2), peripheral location (Figures 3 and 4), and mixed location (Figures 5 and 6). Of the 260 patients, 226 (87%) had central location lesions, and 231 patients (89%) had peripheral location lesions. Exclusive central lesion location occurred in 29 patients (12%), exclusive peripheral location occurred in 34 patients (14%), and mixed central and peripheral location occurred in 197 patients (75%). Lesions located in the lung parenchymal tissue occurred in 15 patients (6%), lesions within pulmonary veins occurred in 99 patients (38%), and mixed lung parenchymal and pulmonary vein lesions occurred in 124 patients (56%). Finally, 82 (32%) of the original 260 patients had lesions within the proximal pulmonary vein and/or entering the left atrium (Figures 2(b), 3(b), 4(b), 5(b), 6(b), 7, and 8).

Out of the 226 patients with centrally located lesions, pulmonary vein lesions occurred in 88 patients (39%) (Figure 2(b)), and mixed lung parenchymal and pulmonary vein lesions occurred in 138 patients (61%) (Figure 1(d)). Bilateral lesion presence was detected in 110 patients (49%), and unilateral presence was detected in 116 patients (51%). Lesions in the superior pulmonary vessel were detected in 195 patients (86%), in the inferior pulmonary vessel in 16 patients (7%), and in both vessels in 15 patients (7%). Among these 226 patients, 93% had lesions located within their superior pulmonary vein (Figures 1(d), 2(b), 5(b), and 6(b)).

**Figure 9:**
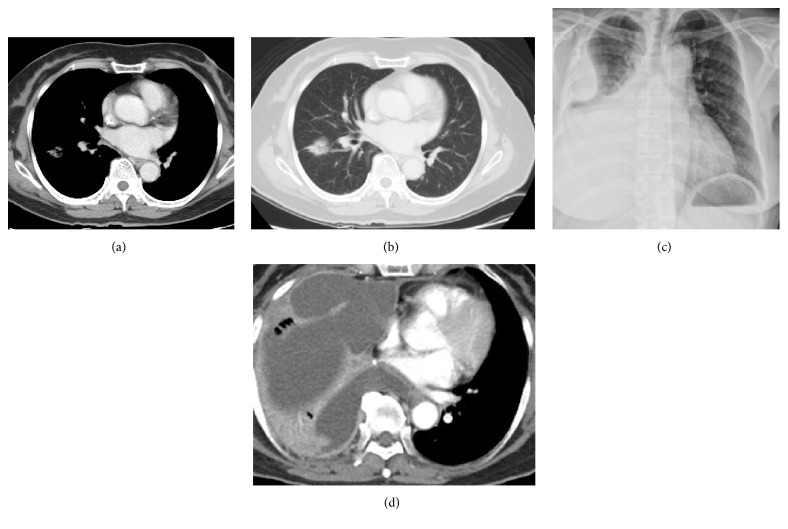
Tumor progression related to pulmonary vein. The patient is a 71-year-old woman with lung adenocarcinoma who underwent a lobectomy. An initial right asymptomatic lung lesion was noted. Two months later she felt dyspnea. A chest CT scan (a) reveals a right lung nodule and a lung window scan (b) shows nodules within right superior vein. Two months later, the tumor had rapidly progressed. A chest X-ray (c) reveals a right lung opacity with right pleural effusion. A CT scan (d) shows right lung parenchymal lesion outside the right superior pulmonary veins with right pleural effusion.

Out of the 231 patients with peripherally located lesions, lesion presence in the pulmonary vein was detected in 87 patients (38%) (Figures 7 and 8), and lesion presence in the mixed lung parenchymal and pulmonary vein locations was detected in 144 patients (62%) (Figures 3(b) and 4(b)). Bilateral lesion presence was detected in 150 patients (65%) and unilateral presence was detected in 81 patients (35%). Lesions in the superior pulmonary vessel were detected in 70 patients (30%), in the inferior pulmonary vessel in 111 patients (51%), and in both vessels in 74 patients (19%). Overall, lesions in the inferior pulmonary vein were detected in 70% of these 231 patients (Figures 4(b), 5(b), and 6(b)).  Figure 9 shows rapid peripheral tumor progression after lung adenocarcinoma two months after a lung lobectomy.

Obvious left atrium extension was found in 59 patients (23%) (Figures 6(b) and 8). All patients presented with nodules in their pulmonary veins (Figures 3(b), 7, 8, and 9).

### 3.3. Treatment Outcome and Survival Data

LMWH therapy was given to 218 PVOS patients, including 113 treated with Fraxiparin (3800 IU or 5700 IU daily) and 95 treated with enoxaparin (6000 IU daily). Intravenous dexamethasone was used on 161 patients. Symptoms were relieved and image improvement occurred in 141 patients (65%), including 135 of those treated with dexamethasone. Of these patients, 48 continued LMWH therapy for secondary prevention.

Sixty-nine patients received alternative therapy, including chemotherapy for 59 patients, targeted therapy for 9 patients, and hormone therapy for 1 patient. Forty-six patients (68%) exhibited disease control. PVOS developed again after disease progression in 32 patients (74%), including 21 who were treated with LMWH therapy for secondary prevention.

Follow-up periods ranged from 1 day to 267 weeks. 13 patients were lost during follow-up; however, 244 patients were followed up until death or up to the present. Three patients survived to the present. Median overall survival time by Kaplan-Meier methods was 5 weeks. The 3-month, 6-month, 1-year, and 2-year survival probabilities were 29%, 14%, 8%, and 2%, respectively.

The predeath status was identified in 234 patients as respiratory failure (*n* = 93; 40%) or loss of consciousness (*n* = 141; 60%). Sixty-nine patients (29%) died of septicemia or febrile neutropenia, including 35 who had respiratory failure and 34 who exhibited a loss of consciousness.

## 4. Discussion

PVOS occurred in 20% of our original 1256 patients who were hospitalized with various malignancies. PVOS encompasses the thromboembolic events that are often associated with other thromboembolic complications. The 260 PVOS patients also exhibited paraneoplastic syndromes that are related to cytokine production [18–20].

This paper demonstrates that pulmonary veins most likely play an important role in tumor progression. This is because tumor/thrombotic lesions within pulmonary veins can spread diffusely. About three-quarters of the PVOS patients in this study had mixed centrally and peripherally located lesions. More than 60% of the patients had tumor/thrombotic lesions that extended into the lung parenchymal tissue, and greater than 20% of patients exhibited obvious lesion extensions into the left atrium of the heart. The anatomy of the superior pulmonary vein and the inferior pulmonary vein differs. More than 90% of central lung lesions originate in the superior pulmonary vein, either bilaterally or unilaterally. About 70% of peripheral lung lesions arise from inferior pulmonary veins, and these are predominantly bilateral (65%).

**Figure 10:**
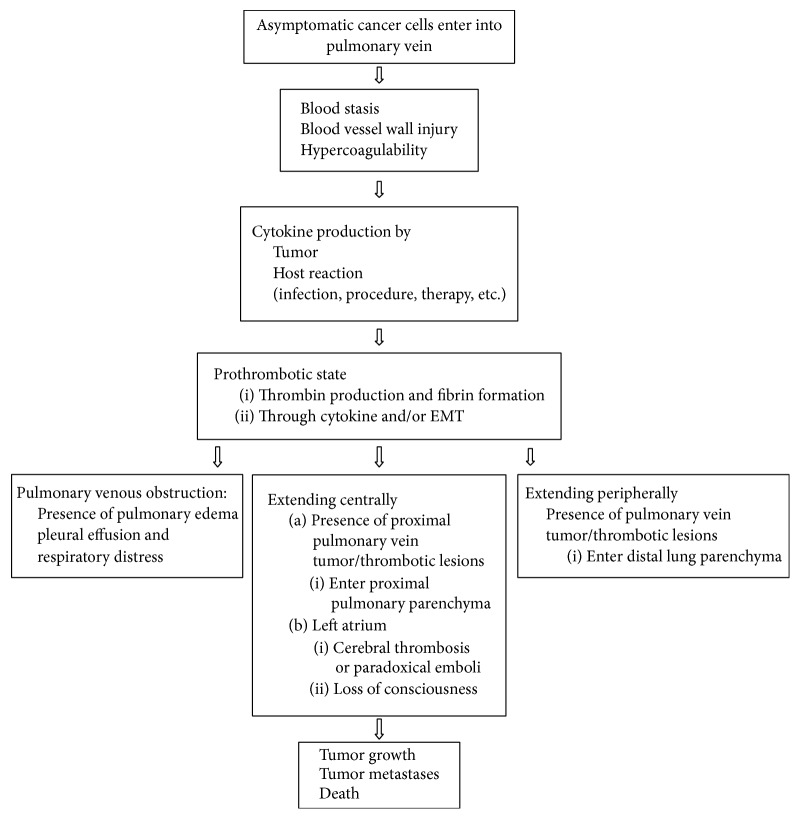
Probable mechanisms of pulmonary vein related to cancer progression.

Figure 10 demonstrates that the probable mechanisms of how pulmonary vein lesions relate to cancer progression. Initially, nodules enter pulmonary veins via tumor metastasis [1]. The cancer cells are found in the tumor-draining veins of patients with non-small-cell lung cancer [3]. They have thrombotic potential and can induce coagulation [4]. Virchow describes three elements, including venous stasis, endothelial injury, and hypercoagulability, that are thought to contribute to venous thromboembolism (VTE) [21, 22]. Once cancer cells enter into and approach the pulmonary vein, blood flow stasis and vascular injury can result. Cancer patients can then enter into a hypercoagulability state. Significant increase of tumor cell abundance in pulmonary venous blood is related to the production of inflammatory cytokines [23, 24]. Thrombin-activated tumor cell adhesion to host cells also enhances tumor cell growth, seeding, and spontaneous metastasis and can stimulate tumor angiogenesis [22–24]. Cytokine production intricately relates both to tumor production and to a host's reaction to infection, chemotherapy, and invasive therapeutic procedures [5, 25, 26]. For example, Reddy et al. reported that pulmonary venous blood sampling can significantly increase the yield of circulating tumor cells in early-stage lung cancer [27].  Figure 9 demonstrates the case of rapid tumor progression after lung adenocarcinoma following a lung lobectomy. Cytokine production related to surgical procedures was probably the main culprit of this phenomenon. Elevated D-dimer or CRP levels have been associated with increased production of cytokines and thrombosis [28, 29].

In more than 20% of our patients, the CT scans allow detection of obvious tumor thrombus lesions in the left atrium. About 45% of these cases were associated with consciousness disturbance. Nearly 60% of the patients died after a loss of consciousness. Tumor/thrombotic lesions can travel from the pulmonary veins through the left atrium and left ventricle and enter the brain circulation [1–3]. However, the pathogenesis is generally parenchymal metastases, rather than vascular thrombotic complications [30]. There are two possible mechanisms that can cause ischemic strokes. One is associated with thromboembolic phenomenon, where hypercoagulability of cerebral veins or arteries occurs. The route of the blockage may be related to the metastatic process. The other mechanism is the association of strokes with paradoxical embolisms [16, 17, 31]. More than 60% of patients had tumor/thrombotic lesions that extended into their lung parenchymal tissue. EMT and inflammatory cytokines have been associated with cancer metastasis and therapy resistance [10–15]. TGF-*β* promotes heterogeneity and drug resistance in cancer patients, where tumor-initiating stem cells often escape cancer therapies [14, 32].

Because of TGF*β* signaling in tumor Initiation, EMT, and metastasis, we can consider using potentially effective therapies to control cancer during the EMT period before tumor resistance develops [10–14, 32]. Treatment is principally aimed at decreasing cytokine and thrombosis production. Cytokines and nuclear factor-Kappa B (NF*κ*B) inhibitors, such as dexamethasone, are used to suppress cytokine formation [33, 34]. LMWH therapy was also reported to downregulate tissue factor expression and activity, by modulating the growth factor receptor-mediated induction of NF*κ*B [34]. In addition, preventing unnecessary procedures and calming patients [35] can reduce cytokine overproduction. Known aggravation factors involved with cytokine production include infection, chemotherapy, and invasive treatment procedures. Adequate fluid supplementation is necessary for maintaining sufficient vital organ perfusion for the brain, coronary artery, and the kidney [4]. LMWH therapy was reported to inhibit plasma thrombin generation [36] and can also inhibit chemotactic migration of cancer cells [37]. Chemokines are also related to tumor progression and metastasis [38]. Maintenance of LMWH therapy is suggested for secondary prevention [39, 40] and probably confers a survival benefit [41, 41, 42]. Underlying disease therapy can be conducted if it is considered useful in overcoming drug resistance; however, it is overall advantageous to avoid rigorous treatment in order to prevent cytokine overproduction [43].

Our study has several important limitations. First, the data was collected from a case cohort study in a single center, mainly from a single physician. Second, the images used in the study, including chest plain films and CT scans, were not conducted simultaneously. Third, the differentiation of pulmonary vein thrombosis, tumor, or mixed type was difficult to judge from the CT scans. Fourth, the loss of consciousness and mental changes related to thromboembolic complications were seldom proven by image study. Fifth, there was an absence of tissue confirmation of the diagnosis by either biopsy or autopsy. Sixth, no cytokine study was conducted.

## 5. Conclusion

Understanding the role of pulmonary veins in cancer progression using CT scans is important. PVOS cancer can enter centrally into the left atrium, which can result in cerebral thromboembolism and eventually lead to death. Cancer may metastasize from pulmonary veins into the lung parenchymal tissue and can result in drug resistance. We suggest that decreasing cytokine production, using LMWH therapy and early treatment of metastatic lung lesions can help PVOS patients.

## Figures and Tables

**Table 1:** Data obtained from 260 cancer patients with pulmonary vein obstruction syndrome (PVOS).

Characteristics	Number of patients (%)
Age (years)	
Median (range)	70 (27–93)
Sex	
Male/female	164/96
Primary sites, number/total hospitalized number (%)	
All patients	260/1256 (20)
Urinary tract	98/416 (20)
Lung	37/130 (27)
Gastrointestine	33/159 (21)
Breast	19/61 (31)
Others	74/490 (15)
Performance status, number/total number (%)	
0-1	75/260 (29)
≥2	185/260 (71)
Associated with other thromboembolic complications, number/total number (%)	
Yes	171/260 (66)
No	89/260 (34)
Associated with other paraneoplastic syndrome, number/total number (%)	
Yes	131/260 (50)
No	129/260 (50)

**Table 2:** Chest CT scan findings relating to the pulmonary veins from 260 cancer patients with PVOS.

Characteristics	Number of patients (%)
By location	
Central location only	29/260 (12)
Peripheral location only	34/260 (14)
Mixed location	197/260 (75)
By organ	
Within pulmonary vein only	99/260 (38)
Both lung and pulmonary vein	161/260 (62)
Central location	226/260 (87)
Central pulmonary vein only	88/226 (39)
Both central lung and pulmonary vein	138/226 (61)
By pulmonary lesions	
Bilateral lung	110/226 (49)
Unilateral lung	116/226 (51)
By pulmonary vein	
Superior pulmonary vein only	195/226 (86)
Inferior pulmonary vein only	16/226 (7)
Superior + inferior pulmonary vein	15/226 (7)
Peripheral location	231/260 (89)
Peripheral pulmonary vein only	87/231 (38)
Both peripheral lung and pulmonary vein	144/231 (62)
By pulmonary lesions	
Bilateral lung	150/231 (65)
Unilateral lung	81/231 (35)
By pulmonary vein	
Superior pulmonary vein only	70/231 (30)
Inferior pulmonary vein only	117/231 (51)
Superior + inferior pulmonary vein	44/231 (19)
Obvious left atrium extent	59/260 (23)
Nodules within pulmonary vein	260/260 (100)
